# Inhibitory and Facilitatory Cueing Effects: Competition between Exogenous and Endogenous Mechanisms

**DOI:** 10.3390/vision3030040

**Published:** 2019-08-22

**Authors:** Alfred Lim, Vivian Eng, Caitlyn Osborne, Steve M. J. Janssen, Jason Satel

**Affiliations:** 1 School of Psychology, University of Nottingham Malaysia, Semenyih 43500, Malaysia; 2 Division of Psychology, School of Medicine, College of Health and Medicine, University of Tasmania, Launceston, Tasmania 7248, Australia

**Keywords:** attention, inhibition of return, saccadic responses, predictive cueing, sensory adaptation, dynamic neural field model

## Abstract

Inhibition of return is characterized by delayed responses to previously attended locations when the cue-target onset asynchrony (CTOA) is long enough. However, when cues are predictive of a target’s location, faster reaction times to cued as compared to uncued targets are normally observed. In this series of experiments investigating saccadic reaction times, we manipulated the cue predictability to 25% (counterpredictive), 50% (nonpredictive), and 75% (predictive) to investigate the interaction between predictive endogenous facilitatory (FCEs) and inhibitory cueing effects (ICEs). Overall, larger ICEs were seen in the counterpredictive condition than in the nonpredictive condition, and no ICE was found in the predictive condition. Based on the hypothesized additivity of FCEs and ICEs, we reasoned that the null ICEs observed in the predictive condition are the result of two opposing mechanisms balancing each other out, and the large ICEs observed with counterpredictive cueing can be attributed to the combination of endogenous facilitation at uncued locations with inhibition at cued locations. Our findings suggest that the endogenous activity contributed by cue predictability can reduce the overall inhibition observed when the mechanisms occur at the same location, or enhance behavioral inhibition when the mechanisms occur at opposite locations.

## 1. Introduction

Using Posner’s traditional cueing paradigm [1], it has been found that reaction time (RT) is typically slower in trials in which the target is presented at a location previously occupied by the cue as compared to when the target is presented at a new location. Such a delay in responses to cued targets, relative to uncued targets, is termed inhibition of return (IOR; [2]) and has been hypothesized as a mechanism that encourages novelty seeking [3] and visual foraging [4]. Slowed RTs to cued as compared to uncued targets are often attributed to IOR, but recent studies suggest that there is more than one inhibitory mechanism (apart from IOR) contributing to such a behavioral consequence [5,6]. Taking into account other inhibitory mechanisms, we thus refer to all behavioral inhibitory effects as inhibitory cueing effects (ICEs), as suggested by Hilchey et al. [5].

In the traditional cueing paradigm, a cue does not convey any spatial information regarding the upcoming target. That is, the target has as much chance of appearing at the cued location as at the uncued location, and, therefore, this paradigm is also referred to as a nonpredictive cueing task. On the other hand, in a predictive cueing paradigm where cues always predict the upcoming target location, inhibition is not observed due to a facilitatory predictive endogenous signal overcoming the inhibitory effects of ICEs (e.g., [7]). Even when the cues are only partially predictive (e.g., 80% predictive of an upcoming target location), behavioral inhibition normally disappears, and facilitation effects are often observed at longer cue‒target onset asynchronies (CTOAs). The predictability of cues can be manipulated by changing the number of cued and uncued trials in an experimental block (e.g., [8,9]). For instance, a cue predictability of 75% in an experimental block with 480 trials can be achieved by having 360 cued trials (i.e., 75% of the 480 trials) and 120 uncued trials (i.e., 25% of the 480 trials). By increasing the chance of a target appearing at the same location as the cue (i.e., predictive cueing), it has been found that ICEs are no longer observable with this manipulation. Instead, facilitatory cueing effects (FCEs), or faster RTs to cued (or valid) as opposed to uncued (or invalid) trials, are often observed, even at long CTOAs (e.g., [3,9,10,11,12]).

### 1.1. Multiple Inhibitory Cueing Mechanisms

The theory that there are multiple inhibitory cueing mechanisms [5,6,13] argues that sensory adaptation, IOR (or direct inhibition), exogenous, and endogenous activity are additive. Sensory adaptation is an input-based inhibitory mechanism that slows responses at a previously attended location and occurs only at short presentation intervals when there is repeated stimulation at the same spatial location, whereas it is thought that direct inhibition of superior colliculus (SC) neurons occurs at longer intervals (i.e., IOR). According to this theory, the endogenous facilitation effect, stemming from the predictability of a cue, is additive with other inhibitory mechanisms (e.g., sensory adaptation and IOR). To further validate this theory, we were interested in the present study in dissociating endogenous facilitation and exogenous inhibition by investigating the effects of cue predictability on the time course of behavioral cueing effects (CEs).

Previous studies have reported mixed results in terms of the influence of cue predictability. For instance, Wright and Richard [9] found ICEs when measured with manual key presses in a nonpredictive condition, but, when the cues were 80% predictive, opposite effects (i.e., FCEs) were observed. In an event-related potential (ERP) study with a cue predictability of 75%, no significant difference in manual RTs to cued and uncued trials was found with a CTOA of 1000 ms [8]. However, although Chica and Lupiáñez [8] did not observe the FCEs reported by Wright and Richard [9] with predictive cueing, ICEs that are commonly found using a traditional cueing paradigm at around 1000 ms CTOAs (e.g., [5,14]) were absent as well. These behavioral results suggest that predictive cueing can influence behavior, and the contradictory results between the two studies could be due to the different CTOAs (i.e., 400 ms vs. 1000 ms) or predictive values used—80% in [9] and 75% in [8]. In another manual detection task comprised of a block of nonpredictive trials followed by a block of 80% predictive trials [15], it was found that ICEs observed in the nonpredictive cueing block were diminished in the predictive cueing block, indicating that ICEs can be masked by endogenous orienting.

In addition, ERP studies have investigated the effects of cue predictability on IOR by using the early visual P1 component as a marker. The P1 component occurs approximately 100 ms after stimulus presentation and has been a major component of investigation in the field of spatial attention [16,17]. It has been interpreted as a sensory gain mechanism that enhances the perceptual processing of stimuli—there are stronger responses for attended-location stimuli compared to ignored-location stimuli [18]. Studies have observed P1 modulations with nonpredictive cues at very short CTOAs, where the amplitude of P1 was enhanced for cued as opposed to uncued trials [19], and reduced for cued as opposed to uncued trials at longer CTOAs (e.g., [11,20,21,22,23,24,25,26]). Using a manual discrimination task with CTOAs of 100, 300, 500, and 700 ms, Doallo et al. [11] investigated the differences in the time course of facilitatory effects induced by the predictability of cues (predictive and nonpredictive). The study found that P1 amplitude on cued trials was reduced at 300, 500, and 700 ms CTOAs with nonpredictive cueing, but only at a 500 ms CTOA with predictive peripheral cueing, suggesting that predictive cueing influences early visual processing, compensating for the inhibitory effects.

### 1.2. Facilitatory and Inhibitory Components

Using a manual discrimination task with CTOAs of 100, 500, and 1000 ms, López-Ramón, Chica, Bartolomeo, and Lupiáñez [27] manipulated cue predictability with three varying conditions: 50% (nonpredictive), 75% (predictive), and 25% (counterpredictive; 75% chance for target to appear at the uncued location). The experiment required participants to pinpoint the target (“x” or “o”) and assess the predictive value of the cue after each block of the experiment. Based on the results of the post-experiment questionnaire, participants were divided into two groups, good estimators and bad estimators, which were analyzed separately. The study found ICEs in the counterpredictive condition and FCEs in the predictive condition. In the nonpredictive condition, ICEs were observed in the bad estimators group at 500 and 1000 ms CTOAs, but not in the good estimators group.

At the time that López-Ramón et al. [27] published their study, the effects that estimation of cue predictability could have on ICEs had not been well examined—although it was hypothesized that this estimation might affect the choice of strategy used by participants. Consequently, it is not clear why an ICE was not found in the nonpredictive condition in [27]. Furthermore, the study included cue predictability as a within-subjects factor, with the nonpredictive block as the first block presented to all participants without counterbalancing. Thus, being exposed to nonpredictive blocks before predictive or counterpredictive blocks could have confounded the effects observed in later blocks.

Taken together, evidence from these studies (i.e., [8,9,11,15]) suggests that the endogenous facilitatory effect of cue predictability may sum additively with inhibitory effects, and they each (endogenous facilitatory effect and inhibitory effect) could take place at different spatial locations—e.g., a facilitatory effect at uncued locations and an inhibitory effect at cued locations in a counterpredictive condition [8]. Thus, we hypothesized that the endogenous facilitation induced by cue predictability is another mechanism that is additive with cue-elicited exogenous inhibitory mechanisms (i.e., sensory adaptation and IOR). This hypothesis suggests that the ICEs observed in traditional cueing paradigms (e.g., [3,5,6]) could be reduced by endogenously orienting attention to cued locations with predictive cueing or further enhanced by endogenously orienting attention to uncued locations with counterpredictive cueing.

The convergence of facilitatory activity (e.g., exogenous and endogenous) and direct inhibition is thought to occur in the intermediate layers of the superior colliculus (iSC) that receive complex visual and cognitive input from various cortical areas [28,29]. The iSC contains a retinotopic motor map for saccade generation, with each location associated with a particular direction and amplitude of a saccade [30]. When a visual stimulus becomes the target for a saccade, clusters of neurons in the iSC that are related to the location of the upcoming saccade increase their activity and trigger a saccade when the activity reaches a threshold level [31]. Using monkey subjects in a predictive cueing task that required a saccadic response to targets, single-cell recordings from the iSC showed enhanced activity at cued locations at a relatively long CTOA of 650 ms [10]. With a shorter CTOA of 250 ms, however, no similar enhanced activity was observed due to sensory adaptation being near its maximal strength and the predictive endogenous signals being too weak to overcompensate for the overall activity loss caused by sensory adaptation. These results indicate that endogenous information contributed by predictive cues reduces the neural activity threshold required to initiate a saccade to the previously cued location, as illustrated by Satel, Wang, Trappenberg, and Klein [32] using a dynamic neural field (DNF) model.

A DNF can describe the statistical average behavior of clusters of neurons with similar receptive fields [33], and can be used to simulate both exogenous and endogenous inputs to the iSC (e.g., [32,34,35]). The model is characterized by short-distance excitation of nearby clusters and long-distance inhibition that captures the behavior of iSC neurons well. DNF models have been demonstrated to be effective at modeling the dynamics of the iSC by deriving parameters from neurophysiological studies in monkeys [35] and reproducing behavioral effects in monkeys and humans. One-dimensional DNF models have been used extensively to model the dynamics of the iSC in the context of modeling visual attention and IOR [13,23,32,35,36], and were later expanded to two-dimensional DNF models to better simulate the iSC (e.g., [6,34]). In particular, Lim et al. [6] demonstrated that ICEs can be explained by the combination of additive facilitatory (i.e., exogenous activity, endogenous activity) and inhibitory components (i.e., sensory adaptation, direct inhibition).

### 1.3. The Present Studies

The aim of the present study was to address the following questions motivated by previous studies: if exogenous activity can reduce the ICEs observed at early CTOAs, can cue-induced predictive endogenous activity (e.g., predictive cueing) also reduce or even outweigh ICEs? If so, what is the time course of such facilitation effects? In addition, based on the proposed theory of the additive nature of facilitatory and inhibitory cueing effects, can we enhance ICEs by manipulating the predictability of a cue (i.e., in a counterpredictive condition)?

We investigated the time course of inhibitory and facilitatory cueing effects with three cue predictabilities (75%, 50%, and 25%) as a between-subjects factor. Because the objective of the present study was to examine the additivity of facilitation and inhibition, peripheral cues were used to induce ICEs. Furthermore, we have not provided information on the predictability of cues or post-test questionnaires, because we reason that this design is a better representation of visual orienting in natural environments in which no predictive values are given, nor are people required to identify the probabilities of any of the visual cues shown.

A series of experiments involving a comparison of the facilitatory and inhibitory effects of cueing as a function of cue predictability and CTOA were conducted along with simulations of the experiments using a two-dimensional DNF model of the iSC. Study 1 used CTOAs of 300, 600, and 900 ms and Study 2 used CTOAs of 900, 1200, and 1500 ms to observe the time course of CEs with 75% (predictive), 50% (nonpredictive), and 25% (counterpredictive) cue predictability.

## 2. Study 1: Short CTOAs

Cue predictability was manipulated between 75% (Exp. 1), 50% (Exp. 2), and 25% (Exp. 3). As such, informative cues (when targets have either 75% or 25% chance of appearing at the same location as cues) were used to endogenously orient the spatial attention of participants prior to target onset. Targets and cues were peripheral onsets throughout all conditions. We hypothesized that in Experiment 1 (75% predictability), the endogenous activity contributed by cue predictability would overcompensate for the ICEs observed in the traditional cueing condition (50% predictability) of Experiment 2. Consequently, we expected to observe either facilitation effects or, at least, nullification of the ICEs observed in the nonpredictive condition. On the other hand, cues with 25% predictability in Experiment 3 would facilitate endogenous orienting to uncued locations, so together with the inhibitory effects generated at cued locations, we should observe relatively stronger ICEs in the counterpredictive condition compared to the nonpredictive condition.

### 2.1. Method

#### 2.1.1. Ethics Statement

The experiments were approved by the Science and Engineering Research Ethics Committee (SEREC) of the University of Nottingham Malaysia (approval number SZ051015) on 7 October 2015. All participants gave written informed consent prior to the experiment.

#### 2.1.2. Participants

In each of the three experiments, 20 students from the University of Nottingham Malaysia participated. The 60 students (39 females and 21 males, mean age 21.3 years, age range 18–27 years) participated in a 45-min session for course credit or monetary compensation (RM10). All participants were naive to the hypotheses of the experiment and reported normal or corrected-to-normal vision.

#### 2.1.3. Stimuli and Apparatus

During testing, participants sat in a dimly lit room with their head positioned on a chin-rest approximately 57 cm away from the display monitor. A 64-bit Microsoft’s Windows 7 (Redmond, WA, USA) computer with a 3.4 GHz CPU and 8 GB of RAM running MATLAB (MathWorks, Natick, MA, USA) scripts was used for stimulus presentation and recording of behavioral data. Stimuli were presented on a 24-inch BenQ (Taipei, Taiwan) gaming monitor. A desktop-mounted eye tracking system (EyeLink 1000 Plus) from SR Research (Ottawa, ON, Canada) was used to monitor participants’ eye movements throughout the experiment with a sampling rate of 1000 Hz. Saccadic response times (SRTs) to targets were recorded using the eye tracker. Drift correction was performed at the beginning of each block of trials.

**Figure 1:**
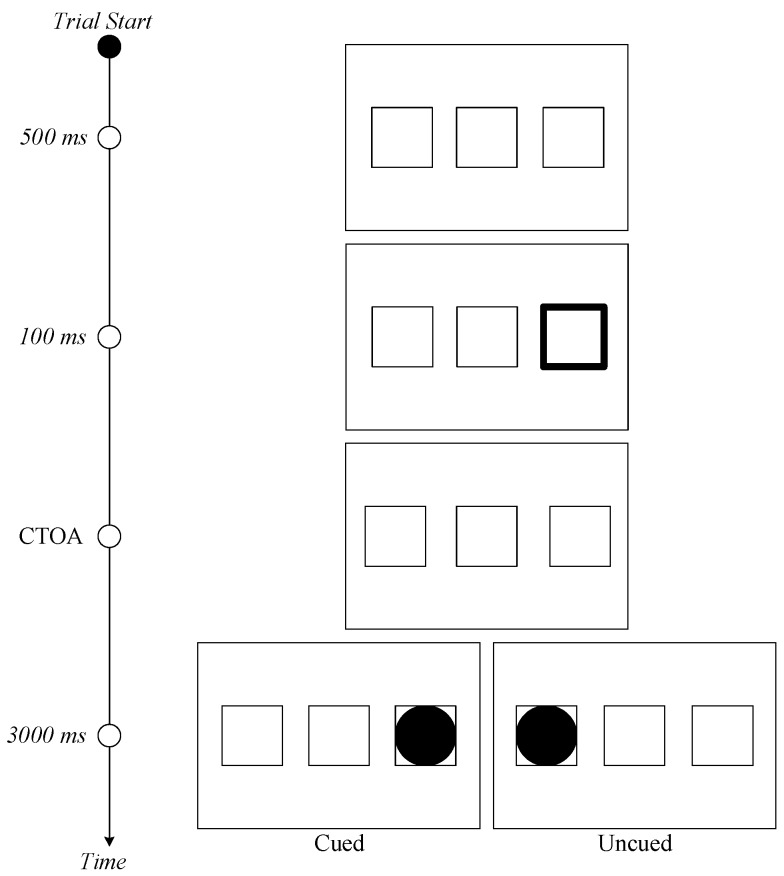
Experimental design used, proceeding temporally from top to bottom. Peripheral cues were presented as a visible amplification of placeholders, followed by peripheral targets as visible filled circles. Participants were asked to ignore cues and make a saccade in response to targets.

The protocol used was a traditional cue‒target paradigm (see Figure 1). Stimuli were displayed against a black background on a computer monitor. A white fixation placeholder was displayed at the center of the screen with two other white placeholders (each measuring 4.5 × 4.5 degrees of visual angle) separated center to center by 9.1° in visual angle, all displayed along the horizontal meridian. The border width of both placeholders initially was set to 1 pixel and later increased to 10 pixels when a placeholder was selected as a cue. The target was a white filled circle (2.4 degrees visual angle in diameter).

#### 2.1.4. Design and Procedure

Each experiment had a 2 (Cueing) × 3 (CTOA) design, with the two variables (Cueing and CTOA) being manipulated within participants. Cueing had two levels (cued and uncued), and CTOA had 3 levels (300, 600, and 900 ms), with 160 trials for each CTOA level. Cueing and CTOA were randomly intermixed within each experimental session. All participants were tested with four blocks of 120 trials (i.e., a total of 480 trials per participant) preceded by 24 practice trials, with rest breaks provided in between blocks (i.e., a total of three breaks per participant). The number of trials per cueing condition depended on the cue predictability of the experiment; Experiment 1 had 360 cued trials (i.e., 75% of all trials), Experiment 2 had 240 cued trials (i.e., 50%), and Experiment 3 had 120 cued trials (i.e., 25%).

The time course of stimulus presentation is shown in Figure 1. Participants began trials by fixating a central placeholder for 500 ms. After the fixation period, a to-be-ignored predictive peripheral cue (thickening of the placeholder’s width) was presented for 100 ms. After a randomly predefined CTOA (300, 600, or 900 ms) had elapsed, a target object represented by a circle was displayed in either the same placeholder as the cue (cued) or the opposite placeholder (uncued) until a saccadic response was made or 3000 ms had passed.

Whereas participants had to remain fixated on the central placeholder when the cue was displayed, they were instructed to make a saccade to the location indicated by the target object as quickly and accurately as possible. SRTs were calculated as the time taken to generate a saccade to target stimuli after their presentation. The criterion for a successful saccadic response was that the saccade fell within at least 3° of visual angle of the center of the targeted location. Additionally, if the participant’s gaze position ever deviated by more than 3° of visual angle from the central box during fixation periods, the trial was abruptly terminated and recycled randomly among the remaining trials.

The experiment lasted on average about 45 min. Each session ended with a debriefing, in which participants were thanked for their contribution and briefly had the IOR phenomenon and the objectives of the study explained to them.

### 2.2. Results

#### 2.2.1. Cue Predictability of 75%

**Figure 2:**
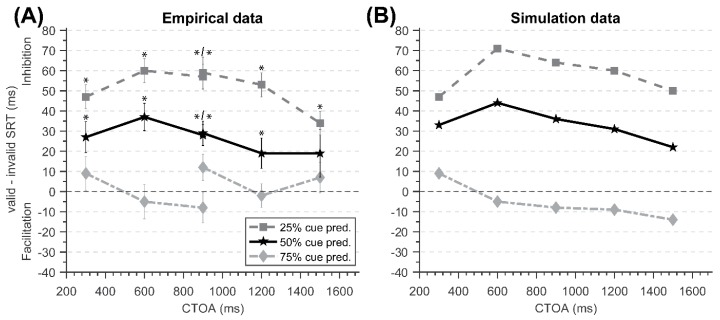
An illustration of the three-way interaction among factors Predictability, Cueing, and CTOA. Square markers denote cueing data with 25% cue predictability, star markers denote cueing data with 50% cue predictability, and diamond markers denote cueing data with 75% cue predictability. (**A**) Empirical data (Studies 1 and 2) with error bars representing the standard error. Asterisks indicate statistically significant differences between the cued and uncued conditions (two-tailed, paired-samples *t*-tests, *p* < 0.05). (**B**) Simulation data.

In the first experiment, cues accurately identified the upcoming location of the target 75% of the time. All participants scored at least 99.16% accuracy (the mean was 99.78%). After excluding incorrect trials (0.22% of all trials), anticipatory responses (SRTs < 2.5 Median Absolute Deviation (MAD—used as a robust measure of variance that is less sensitive to outliers compared to standard deviation [37]) units; 1.09%) and long outliers (SRTs > 2.5 MAD units; 7.77%), statistical analyses were performed on the remaining 90.92% of all trials. Difference scores (cued‒uncued) of correct SRTs for each condition are presented in Table 1 and Figure 2.

All correct SRTs were submitted to a 2 (Cueing) × 3 (CTOA) repeated-measures ANOVA. There was a significant main effect of CTOA [*F*(2, 38) = 40.45, *p* < 0.001, *MSE* = 589.17, *η*^2^ = 0.68], where SRTs became faster as CTOA increased due to temporal predictability, starting at a CTOA of 300 ms (*M* = 324.89), followed by 600 ms (*M* = 294.70), and 900 ms (*M* = 281.70). However, the main effect of Cueing [*F*(1, 19) = 0.05, *p* = 0.821, *MSE* = 1138.02, *η*^2^ = 0.01] did not attain significance, which was confirmed by planned two-tailed paired-samples *t*-tests: 300 ms [CE = −8.82, *t*(19) = 1.33, *p* = 0.198, *d* = 0.30], 600 ms [CE = 5.08, *t*(19) = −0.73, *p* = 0.472, *d* = 0.16], and 900 ms [CE = 7.97, *t*(19) = −0.94, *p* = 0.360, *d* = 0.21]. The interaction involving Cueing and CTOA was non-significant as well [*F*(2, 38) = 3.21, *p* = 0.052, *MSE* = 251.36, *η*^2^ = 0.14].

#### 2.2.2. Cue Predictability of 50%

In the second experiment, cues were nonpredictive and the target appeared with equal probability at either the cued or uncued location. All participants scored at least 99.17% accuracy (*M* = 99.88%). After excluding incorrect trials (0.13%), anticipatory responses (0.83%), and slow outliers (6.06%), statistical analyses were performed on the remaining 92.98% of trials.

All correct SRTs were submitted to a 2 (Cueing) × 3 (CTOA) repeated-measures ANOVA. There was a significant main effect of Cueing [*F*(1,19) = 39.50, *p* < 0.001, *MSE* = 725.73, *η*^2^ = 0.68], with SRTs being slower in the cued condition (*M* = 279.02) than in the uncued condition (*M* = 248.64). Two-tailed paired-samples *t*-tests revealed that all ICEs observed were statistically significant: 300 ms [CE = 27.93, *t*(19) = −3.86, *p* = 0.001, *d* = 0.86], 600 ms [CE = 36.94, *t*(19) = −7.94, *p* < 0.001, *d* = 1.77], and 900 ms [CE = 27.87, *t*(19) = −6.21, *p* < 0.001, *d* = 1.39]. A main effect of CTOA was also observed [*F*(2,38) = 91.92, *p* < 0.001, *MSE* = 334.16, *η*^2^ = 0.83], where SRTs became faster as CTOA increased, starting at a CTOA of 300 ms (*M* = 295.94), followed by 600 ms (*M* = 250.95), and 900 ms (*M* = 245.44). There was no interaction between Cueing and CTOA [*F*(2,38) = 2.53, *p* = 0.093, *MSE* = 107.59, *η*^2^ = 0.12].

#### 2.2.3. Cue Predictability of 25%

In the third experiment, cues accurately identified the upcoming location of the target 25% of the time (counterpredictive). All participants scored at least 97.94% accuracy (*M* = 99.77%). After excluding incorrect trials (0.23%), anticipatory responses (0.88%), and slow outliers (6.93%), statistical analyses were performed on the remaining 91.96% of all trials.

All correct SRTs were submitted to a 2 (Cueing) × 3 (CTOA) repeated-measures ANOVA. There was a significant main effect of Cueing [*F*(1,19) = 232.54, *p* < 0.001, *MSE* = 383.58, *η*^2^ = 0.92], with SRTs being slower in the cued condition (*M* = 292.25) than in the uncued condition (*M* = 237.33). Two-tailed paired-samples *t*-tests revealed that all ICEs observed were statistically significant: 300 ms [CE = 47.39, *t*(19) = −11.24, *p* < 0.001, *d* = 2.51], 600 ms [CE = 60.00, *t*(19) = −15.02, *p* < 0.001, *d* = 3.36], and 900 ms [CE = 56.20, *t*(19) = −8.39, *p* < 0.001, *d* = 1.88]. There was also a main effect of CTOA [*F*(2,38) = 45.89, *p* < 0.001, *MSE* = 341.09, *η*^2^ = 0.71], with SRTs becoming faster as CTOA increased, starting at a CTOA of 300 ms (*M* = 274.79), followed by 600 ms (*M* = 244.41) and 900 ms (*M* = 234.62). There was no interaction between Cueing and CTOA [*F*(2,38) = 2.08, *p* = 0.139, *MSE* = 201.46, *η*^2^ = 0.10].

#### 2.2.4. Cue Predictability of 75% vs. 50% vs. 25%

Finally, to compare the results of the three experiments, we conducted a three-way repeated-measures ANOVA in which Predictability was a between-subjects factor and Cueing and CTOA were within-subjects factors. There was a main effect of Predictability [*F*(2, 57) = 7.26, *p* = 0.002, *MSE* = 7154.60, *η*^2^ = 0.20] showing that responses in the counterpredictive cueing experiment (with 25% cue predictability; *M* = 251.22) were fastest, followed by the nonpredictive cueing experiment (with 50% cue predictability; *M* = 264.05) and then the predictive cueing experiment (with 75% cue predictability; *M* = 300.43).

More importantly, we also observed an interaction between Predictability and Cueing [*F*(2,57) = 28.36, *p* < 0.001, *MSE* = 749.11, *η*^2^ = 0.50], suggesting that the size of the cueing effect (i.e., the difference between responses to cued and uncued trials) changes with cue predictability. To decompose the interaction, we conducted separate two-way ANOVA tests for each pair of predictabilities. These ANOVA tests revealed that the Cueing × Predictability interaction was significant between predictabilities of 75% and 50% [*F*(1,38) = 14.01, *p* < 0.001, *MSE* = 931.87, *η*^2^ = 0.30], 75% and 25% [*F*(1,38) = 55.63, *p* < 0.001, *MSE* = 760.80, *η*^2^ = 0.59], and 50% and 25% [*F*(1,38) = 15.08, *p* < 0.001, *MSE* = 554.65, *η*^2^ = 0.28].

### 2.3. Discussion

We interpreted our behavioral results in terms of a multiple mechanisms theory of ICEs—sensory adaptation is generated at early CTOAs with repeated stimulation, and direct inhibition at late CTOAs. These inhibitory components are additive with the neural activity associated with attentional orienting in the iSC and are considered as facilitatory components. The two types of orienting mechanisms that arise in the spatial orienting paradigm are exogenous and endogenous [1]. Exogenous (bottom-up) orienting is triggered by sensory information from the physical world that attracts our visual attention, whereas endogenous (top-down) orienting is involved in seeking and extracting sensory information, and is driven voluntarily by our knowledge, expectations, and goals (for a review, see [38]). Depending on the design of the experiment, the effects of either one or both of these orienting mechanisms can be observed [14]. For instance, cue predictability can be increased to induce endogenous orienting before target onset, granting endogenous predictability to the cues. In the paradigm used here, exogenous orienting is induced at the onset of each stimulus given their saliency, and endogenous orienting is induced by targets and information related to the onset of targets (e.g., cue predictability).

When a cue is informative, FCEs are often seen at long CTOAs in manual response tasks (e.g., [9,11,39]) because cue predictability induces an endogenous orienting process, overcompensating for the ICEs. With these results in mind, we hypothesized that FCEs would be observed in the experiment when cues were predictive (i.e., 75% cue predictability). However, although overall the participants responded faster to cued than to uncued targets, no significant FCEs were found at any of the three CTOAs.

When compared to the significant ICEs across all CTOAs with 50% cue predictability, these null findings with 75% cue predictability suggest that the FCEs stemming from cue predictability compensated (but did not overcompensate) for the ICEs in the predictive cueing task, leading to stronger ICEs with 50% than with 75% cue predictability. Similar nullification of manual ICEs was observed in the behavioral results obtained by Chica and Lupiáñez [8]. Although their ERP results showed P1 reductions for cued versus uncued trials with 75% cue predictability at CTOA of 1000 ms, they observed no behavioral cueing effect. Furthermore, P1 reductions were observed with both 50% and 75% cue predictability in [8], suggesting that the inhibition effect induced by cue onset cannot be eliminated from cued locations even when cues are spatially predictive of a target appearance at their same spatial location.

Similarly, when comparing the results of the counterpredictive to the results of the nonpredictive experiment, the ICEs observed with a cue predictability of 25% were stronger at all CTOAs as compared to the ICEs observed with a cue predictability of 50%. These findings suggest that the ICEs stemming from cue predictability add to the ICEs that occur when the cues are uninformative. ICEs that were caused by inhibition at the cued locations with 50% cue predictability were further enhanced with 25% cue predictability due to facilitation at cued locations. We infer that this outcome could be due to endogenous activity buildup at uncued locations, because there is a higher chance for target onset at the uncued location with 25% cue predictability. This buildup is different from when cues had a predictability of 75%, which led to facilitation at cued instead of uncued locations. From these results, we conclude that the onset location of inhibitory effects depended on cued locations and the onset location of facilitatory effects can be manipulated by changing the predictability of cues—facilitation at cued locations with 75% cue predictability and uncued locations with 25% cue predictability. These effects are argued to be additive in the iSC in a winner-take-all manner, with the resultant behavioral cueing effects depending on the sum of all effects at the respective cued and uncued locations.

Based on the observations of a single monkey subject by Bell and Munoz [10], there was a trend toward a saccadic FCE (faster SRTs to cued targets) as CTOA increased from 250 to 650 ms in a task with 80% cue predictability, suggesting that the facilitation effect of cue predictability may become stronger over time. However, the results were inconclusive, because the trend was not observed in the other monkey subject that showed ICEs at both short and long CTOAs. Our theory of multiple additive cueing mechanisms suggests that cue predictability should contribute sufficient endogenous facilitatory activity at the predicted location to overcompensate for the inhibitory effects at cued locations and, as a result, lead to FCEs. However, our results demonstrated that the endogenous activity contributed by 75% cue predictability was not enough to produce behavioral facilitation (i.e., faster SRTs in cued as opposed to uncued trials). Although trending towards an FCE as CTOA increases, this trend could be due to either the buildup of facilitation contributed by cue predictability, the dispersion of inhibition of sensory adaptation, or both.

In a previous empirical and computational study [6], we suggested that sensory adaptation and direct inhibition were both active at around 600 ms post-cue. This hypothesis is now further supported by the strongest ICE of 36.94 ms observed here being at a 600-ms CTOA with 50% cue predictability. Additionally, lingering cue-elicited activity that takes time to disperse completely can reduce the ICE observed at a 300-ms CTOA by contributing to the neuronal activity buildup at cued locations in the iSC. Because the cueing effects observed at the short CTOAs of 300, 600, and 900 ms can be affected by both cue-induced exogenous activity and sensory adaptation, the facilitatory effects of cue predictability could be overshadowed by these other components that were active at that time. In the experiment with 75% cue predictability, especially, the evidence was insufficient to determine whether the trend towards FCEs was due to the facilitation effects of cue predictability requiring time to build up, or the dispersion of sensory adaptation over time. To be able to dissociate and to better understand the time course of the endogenous facilitatory component and inhibitory mechanism—cue predictability and sensory adaptation—we conducted a follow-up study with longer CTOAs of 900, 1200, and 1500 ms, a situation in which direct inhibition is likely the only active inhibitory component.

## 3. Study 2: Long CTOAs

With CTOAs of 300, 600, and 900 ms, it was difficult to dissociate endogenous facilitation effects from cueing effects due to multiple other components being present and active at those short CTOAs. In this study, we therefore used longer CTOAs of 900, 1200, and 1500 ms, as opposed to the shorter CTOAs of Study 1 (300, 600, and 900 ms), to further investigate the time course of cue predictability facilitation without the presence of the inhibitory effects of sensory adaptation. Three experiments were conducted in this follow-up study, each with a different cue predictability: 75% (predictive; Exp. 4), 50% (nonpredictive; Exp. 5), and 25% (counterpredictive; Exp. 6).

### 3.1. Method

#### 3.1.1. Ethics Statement

The experiments were approved by the Social Sciences Human Research Ethics Committee (HREC) of the University of Tasmania (approval number H0016538) on 8 May 2017. All participants gave written informed consent prior to the beginning of the experiment.

#### 3.1.2. Participants

One participant was excluded from the analyses due to extreme variation in their overall reaction times (*SD* = 191.27; the next highest *SD* was 117.92) and subsequently replaced.

Three groups of 20 students from the University of Tasmania, Australia, participated in each of the three experiments. The 60 students (43 females and 17 males, mean age 25.6 years, age range 18–58 years) participated in a 45-min session for course credit or monetary compensation (15 AUD). All participants were naive to the hypotheses of the experiment and reported normal or corrected-to-normal vision.

#### 3.1.3. Stimuli and Apparatus

Whereas the stimuli of Study 2 were identical to the ones of Study 1, there were some minor differences in the apparatus that was used. During testing, participants sat in a dimly lit room with their heads positioned approximately 65 cm away from the display monitor. A 64-bit Windows 10 computer with a 3.4 GHz CPU and 8 GB of RAM running MATLAB scripts was used for stimulus presentation and recording of behavioral data. Stimuli were presented on a 27-inch BenQ gaming monitor. A desktop-mounted eye tracking system (EyeLink 1000 Plus) from SR Research was used to monitor participants’ eye movements throughout the experiment at a sampling rate of 500 Hz. Saccadic response times to targets were recorded using the eye-tracker. Drift correction was performed at the beginning of each block of trials.

#### 3.1.4. Design and Procedure

Except for the CTOAs, the design and procedure of Study 2 were identical to those of Study 1. Each experiment had a 2 (Cueing) × 3 (CTOA) design, with the two variables (Cueing and CTOA) being manipulated within participants. The following factors were used: Cueing (with two levels: cued and uncued) and CTOA (with three levels: 900, 1200, and 1500 ms). All participants were tested with four blocks of 120 trials (i.e., a total of 480 trials per participant) preceded by 24 practice trials, with rest breaks provided in between blocks (i.e., a total of three breaks per participant). Number of trials per cueing condition depended on the cue predictability of the experiment, in which Experiment 4 had 360 cued trials (i.e., 75% of all trials), Experiment 5 had 240 cued trials (i.e., 50%), and Experiment 6 had 120 cued trials (i.e., 25%).

### 3.2. Results

#### 3.2.1. Cue Predictability of 75%

All participants scored at least 99.28% accuracy (*M* = 99.84%). After excluding incorrect trials (0.14%), anticipatory responses (0.36%), and slow outliers (6.76%), statistical analyses were performed on the remaining 92.74% of all trials. Difference scores (cued‒uncued) of SRTs for each condition are presented in Table 2 and Figure 2.

All correct SRTs were submitted to a 2 (Cueing) × 3 (CTOA) repeated-measures ANOVA. There was a significant main effect of CTOA [*F*(2,38) = 41.60, *p* < 0.001, *MSE* = 306.74, *η*^2^ = 0.69], where SRTs became faster as CTOA increased, starting at a CTOA of 900 ms (*M* = 297.22), followed by 1200 ms (*M* = 265.76) and 1500 ms (*M* = 262.03). However, there was no main effect of Cueing [*F*(1,19) = 1.15, *p* = 0.297, *MSE* = 844.11, *η*^2^ = 0.06], which was confirmed by planned two-tailed paired-samples *t*-tests: 900 ms [CE = 12.19, *t*(19) = −1.71, *p* = 0.103], 1200 ms [CE = −2.67, *t*(19) = 0.43, *p* = 0.674, *d* = 0.10], and 1500 ms [CE = 7.56, *t*(19) = −1.36, *p* = 0.190, *d* = 0.30]. The interaction between Cueing and CTOA was non-significant as well [*F*(2,38) = 3.18, *p* = 0.053, *MSE* = 181.61, *η*^2^ = 0.14].

#### 3.2.2. Cue Predictability of 50%

All participants scored at least 98.77% accuracy (*M* = 99.94%). After excluding incorrect trials (0.06%), anticipatory responses (0.36%), and slow outliers (7.57%), statistical analyses were performed on the remaining 92.01% of trials.

A 2 (Cueing) × 3 (CTOA) repeated-measures ANOVA indicated there was a significant main effect of Cueing [*F*(1,19) = 49.92, *p* < 0.001, *MSE* = 292.91, *η*^2^ = 0.72], as a result of slower SRTs in the cued condition (*M* = 285.25) than in the uncued condition (*M* = 264.37). Planned two-tailed paired-samples *t*-tests indicated that the ICEs observed at all CTOAs were significant: 900 ms [CE = 29.47, *t*(19) = −7.25, *p* < 0.001], 1200 ms [CE = 18.65, *t*(19) = −3.84, *p* = 0.001], and 1500 ms [CE = 18.12, *t*(19) = −5.05, *p* < 0.001]. There was also a main effect of CTOA [*F*(2,38) = 48.85, *p* < 0.001, *MSE* = 395.09, *η*^2^ = 0.72], where SRTs became faster as CTOA increased, starting at a CTOA of 900 ms (*M* = 299.46), followed by 1200 ms (*M* = 267.14) and 1500 ms (*M* = 257.82). The interaction involving Cueing and CTOA was also significant [*F*(2,38) = 3.48, *p* = 0.041, *MSE* = 118.10, *η*^2^ = 0.15], as a result of weaker ICEs as CTOA increased.

#### 3.2.3. Cue Predictability of 25%

All participants scored at least 99.55% accuracy (*M* = 99.92%). After excluding incorrect trials (0.07%), anticipatory responses (0.56%), and slow outliers (7.42%), statistical analyses were performed on the remaining 91.95% of all trials. A 2 (Cueing) × 3 (CTOA) repeated-measures ANOVA indicated there was a significant main effect of Cueing [*F*(1,19) = 116.10, *p* < 0.001, *MSE* = 617.83, *η*^2^ = 0.86], as a result of slower SRTs in cued conditions (*M* = 309.11) than in uncued conditions (*M* = 259.81). Planned two-tailed paired-samples *t*-tests indicated that the cueing effects observed at all CTOAs were significant: 900 ms [CE = 59.09 ms; *t*(19) = −9.83, *p* < 0.001, *d* = 2.20], 1200 ms [CE = 53.00 ms; *t*(19) = −10.51, *p* < 0.001, *d* = 2.35], and 1500 ms [CE = 34.60 ms; *t*(19) = −5.80, *p* < 0.001, *d* = 1.30]. There was also a main effect of CTOA [*F*(2,38) = 87.73, *p* < 0.001, *MSE* = 317.87, *η*^2^ = 0.82], where SRTs became faster as CTOA increased, starting at a CTOA of 900 ms (*M* = 298.81), followed by 1200 ms (*M* = 262.87) and 1500 ms (*M* = 255.40). The interaction between Cueing and CTOA was also significant [*F*(2,38) = 9.20, *p* < 0.001, *MSE* = 176.63, *η*^2^ = 0.33], as a result of weaker ICEs as CTOA increased.

#### 3.2.4. Cue Predictability of 75% vs. 50% vs. 25%

Finally, to compare the results of the three experiments, we conducted a three-way repeated-measures ANOVA in which Predictability was a between-subjects factor and Cueing and CTOA were within-subjects factors. Although there was no main effect of Predictability [*F*(2,57) = 0.38, *p* = 0.684, *MSE* = 11,399.51, *η*^2^ = 0.01], we observed an interaction effect between Predictability and Cueing [*F*(2,57) = 24.40, *p* < 0.001, *MSE* = 584.95, *η*^2^ = 0.46], showing that the differences in SRT between cued and uncued trials became larger as cue predictability became lower. To decompose the interaction, we conducted separate two-way ANOVA tests for each pair of predictabilities. These ANOVA tests revealed that the Cueing X Predictability interaction was significant between predictabilities of 75% and 50% [*F*(1,38) = 7.09, *p* = 0.011, *MSE* = 568.51, *η*^2^ = 0.16], 75% and 25% [*F*(1,38) = 38.31, *p* < 0.001, *MSE* = 730.97, *η*^2^ = 0.50], and 50% and 25% [*F*(1,38) = 23.69, *p* < 0.001, *MSE* = 455.37, *η*^2^ = 0.38].

### 3.3. Discussion

Similar patterns of ICEs were observed in Study 2 for all cue predictabilities. In the predictive experiment (75% cue predictability), no significant cueing effects—either facilitatory nor inhibitory—were found at long CTOAs. These outcomes are consistent with those in Study 1, where we reasoned that the FCEs induced by predictive cueing effectively canceled out the ICEs, thus resulting in no overall observed cueing effect. This interpretation was again supported by the presence of the ICEs in the nonpredictive experiment (50% cue predictability).

In the experiment with 25% cue predictability, the ICEs observed at all three CTOAs were enhanced compared with those in the nonpredictive condition, presumably as a result of the facilitation of uncued targets due to counterpredictive cueing. Importantly, we observed that overall ICEs decreased as CTOA increased from 900 to 1500 ms that were not observed in Study 1, in which the strongest ICE was observed at 600 ms CTOA. A similar trend to the FCEs observation was also seen in the 50% cue predictability condition, suggesting that the general trend for small ICEs at long CTOAs observed across 25% and 50% cue predictability conditions was caused by the gradual decline in inhibition over time. The degradation of the late inhibitory component, direct inhibition, had led to the overall declination of cueing effects as CTOA increased. A similar trend was not observed consistently in Study 1 because late direct inhibition was not active at short CTOAs, and the function of early sensory adaptation’s inhibitory effects was masked by other facilitatory (e.g., cue-induced exogenous activity) components. In Study 2, however, early exogenous activity was no longer present in the iSC and sensory adaptation had likely dispersed, causing the change of direct inhibition to be observable behaviorally at these longer CTOAs.

It is unlikely that the cue predictability facilitation effects got stronger over time because the cueing effects observed at 900 ms CTOA in the 75% cue predictability condition did not converge across the two studies (short and long CTOAs). With inhibition nullified by the facilitatory effects of 75% cue predictability, the change in cue predictability facilitation over time became evident compared to 25% cue predictability condition, in which the time course of the facilitatory effects of cue predictability were likely masked by the effects of multiple inhibitory mechanisms (i.e., sensory adaptation and direct inhibition). In accordance with the theory that we currently favor to account for the present findings, the facilitation effects of cue predictability are additive, along with the other inhibitory and facilitatory components. That said, the inhibitory components that were present at cued locations partially compensated for the facilitation effects of 75% cue predictability, nullifying the ICEs that were observed with 50% cue predictability.

However, this does not explain the disparate cueing effects between the two studies observed at 900 ms CTOA with 75% cue predictability. One possible explanation for these findings is the variance in the ability to estimate cue predictability between the two group of participants. In a series of experiments conducted by López-Ramón et al. [27], it was found that the F/ICEs observed between good and bad estimators can differ in magnitude. With 75% cue predictability, cueing effects of −39 ms and −7 ms were observed in good estimators and bad estimators, respectively, at 1000 ms CTOA. Although we did not survey the estimation of participants, we found a cueing effect difference of 20 ms between the two experiments at 900 ms CTOA despite the similar experimental design used, only with a different range of CTOAs. We therefore reasoned that the disparity in cueing effects was likely caused by the between-subjects variability in estimating cue predictability.

## 4. Simulations

In a predictive cueing paradigm, where cues always predict the upcoming target location, inhibition is not observed due to a facilitatory predictive endogenous signal overcoming the inhibitory effects of ICEs [7]. Even when the cues are only partially predictive (e.g., 80% predictive of upcoming target location), behavioral inhibition normally disappears, and facilitation effects are observed at longer CTOAs. For example, using monkey subjects in such a paradigm, Bell and Munoz [10] showed facilitation at a relatively long CTOA of 650 ms. With shorter CTOAs, however, inhibition remains observable due to sensory adaptation being near its maximal strength and the predictive endogenous signal presumably still being quite weak. These results indicate that the endogenous information contributed by the predictive cues effectively reduces the neural activity threshold required to initiate a saccade to the previously cued location [32].

A previous computational study by Satel et al. [32] simulated the cue predictability facilitation effects in the iSC by transmitting a growing endogenous input to cued locations. Although the simulated behavioral and neurophysiological results were in agreement with the monkey data reported by Bell and Munoz [10], the model failed to generalize the results to the present study. The CEs observed in the two predictive cueing experiments with different CTOAs, although declining, did not converge at 900 ms CTOA. Our behavioral results demonstrated that the facilitation effects contributed by cue predictability do not grow indefinitely; instead, temporal predictability needs to be implemented in the model to account for faster SRTs as CTOA increases and the SRT trend observed in the experiments.

Gabay and Henik [40] demonstrated that the time course of CEs was modulated by temporal information due to the conditional probability of target onset increasing with CTOA. Most importantly, temporal predictability causes the SRTs to decrease as CTOAs increase. We hypothesized that temporal predictability is another facilitatory endogenous component (referred to as the “temporal predictive component” from here onwards) that is different from the predictive endogenous component caused by cue predictability. Unlike cue predictability, the facilitation effects of temporal predictability should be present at both cued and uncued locations due to temporal predictability providing no spatial information about the target appearance, facilitating responses to targets regardless of their onset location.

The model used in the present study has the same parameters as our previous study [6], which has the following components: sensory adaptation, direct inhibition, temporal predictability endogenous signal, target-induced move endogenous signal, and cue-induced exogenous signal. We also added a cue predictability endogenous signal to the existing model to simulate the effects of cue predictability. As the multiple inhibitory cueing mechanisms theory suggests, all inhibitory components (e.g., sensory adaptation, direct inhibition) and facilitatory components (e.g., cue-induced exogenous signal, cue predictability endogenous signal) are additive in the DNF model of the iSC.

The objective of the simulations was to support the inferences made from the behavioral results gathered: (1) greater ICEs were observed at a CTOA of 600 ms with 25% and 75% cue predictabilities due to sensory adaptation and direct inhibition; (2) greater ICEs across all CTOAs with 25% compared to 50% cue predictabilities due to cue predictability endogenous activity at uncued locations; (3) sensory adaptation and direct inhibition were both active even with 75% cue predictability, but their effects were overcompensated for by cue predictability endogenous activity at cued locations; and (4) the cueing effects trend observed at late CTOAs with 25% and 50% predictabilities was caused by the degradation of direct inhibition and not by the cue predictability endogenous activity, which grows indefinitely.

### 4.1. Model Architecture

The two-dimensional DNF model simulates the temporal average of individual neurons in terms of a space- and time-continuous distribution of its neural activation, *u*(*x, y, t*), which describes the average activity of a cluster of neurons at a two-dimensional location (*x, y*) at time *t*. The dynamics of the activation function *u*(*x, y, t*) are given as follows:

$$\tau \frac{du(x,y,t)}{dt}=-u(x,y,t)+\int_{a}\int_{b}w(x,y,a,b)r(a,b,t)dadb+I{\left(x,y,t\right)}^{\mathrm{ext}},$$

where *τ* = 90 is a time constant that determines the time scale of the dynamics, and *u*(*x, y, t*) is a leaky integrator unit that describes the leaky integrator characteristics of local neuron dynamics.

The weight matrix, *w*, represents the connection strengths between nodes at different locations in a Mexican hat pattern of lateral interactions, short-distance excitatory, and long-distance inhibitory [41]. Hence, the connection weight between a node at location (*x, y*) and location (*a, b*) is determined by:

$$w(x,y,a,b)=A_{w}e^{-[{\left(x-a\right)}^{2}+{\left(y-b\right)}^{2}]/2\sigma_{w}^{2}}-C,$$

where *A* = 1, *C* = 1/5, *σ* = *pi*/5 to match with existing empirical results. Input to the model, *I*(*x, y, t*)^ext^, represents the external input to the field at location (*x, y*) at time *t*.

The activity of a node at a location, *r*(*x, y, t*), is defined by the convolution of a Gaussian kernel with a nonlinear sigmoid threshold function given by:

$$r(x,y,t)=1/\left(1+e^{-\beta u}\right),$$

where *β* = 0.08 represents the slope of the sigmoid that determines whether a node’s activity is close enough to the threshold to contribute to the activation dynamics.

Since it is more likely for a target to appear as each of the possible CTOAs elapses, participants can make use of this temporal information to predict of target onset. This effect is termed temporal predictability and has been found to cause faster response times to targets as CTOA elapses in a task [40,42]. To account for temporal predictability in our model, a function that serves as an endogenous signal multiplier is defined as follows:

$$p(t)=a[t/\left(b+c\right)]e^{1-[t/\left(b+c\right)]},$$

where *a* denotes the largest possible multiplier, *b* is the time when the function reaches its maximal multiplier as defined by *a*. The function is further scaled by a cortical processing delay, variable *c*. Parameters of *p*(*t*) used in our model are *a* = 0.05, *b* = 700, and *c* = 120, specifying that the function reaches its maximum multiplier of 0.05 at time 700 + 120 ms post-cue.

### 4.2. Model Parameters

For consistency and to allow for general comparisons across studies, the parameters of the model used in the present study are similar to those in our previous study (see [6]), which were determined based on behavioral and neurophysiological data (e.g., [35,43,44]), with an added predictive endogenous input (*I_endoPred_*) to simulate the facilitation effect contributed by cue predictability and a new decay constant for direct inhibition to simulate the gradual dispersion of inhibition at long CTOAs.

Behavioral results were simulated according to the multiple mechanisms theory of cueing effects, which comprises two inhibitory (i.e., sensory adaptation and direct inhibition) and two facilitatory (i.e., exogenous and endogenous) components. Endogenous signals simulated in the model can be further broken down into different types depending on the cause of such activity; these include: target-induced move signal (*I_endoMove_*), cue predictability-induced endogenous signal (*I_endoPred_*), and temporal predictive endogenous signal (*I_endoTemp_*). Each input type (*I_x_*) has different trigger conditions and is predefined with an initial strength value and a growth, or decay, constant (*t_x_*). For instance, the task-relevant endogenous move input (*I_endoMove_*) induced by target onset grows indefinitely until any node reaches the threshold activity for saccade initiation. On the other hand, the predictability-induced endogenous input (*I_endoPred_*) is only triggered upon a predictive or counterpredictive cue onset and grows until 620 ms post-cue onset, then is sustained indefinitely at its final strength value. All exogenous and endogenous inputs were sent to the model with a brief delay of 70 and 120 ms, respectively, to simulate sensory and cortical processing delays (e.g., [32,35]).

Sensory adaptation begins with the onset of the cue, growing to its strongest attenuation strength at 450 ms, and finally, decays over time until it disperses completely at 750 ms post-cue. The other inhibitory mechanism, direct inhibition (i.e., IOR), was implemented as a negative input, *I_di_*, that began with a strength of 0.07 units, and increased gradually according to a pre-defined growth constant of, *t_diGrowth_ =* 1/140, up to a maximum strength of 1.14 units at 950 ms post-cue. At 1300 ms post-cue, direct inhibition (*I_di_*) began to decay gradually at, *t_diDecay_ =* −1/1000, until complete dispersion. Fixations throughout a trial are represented by a sustained input signal with a strength of 30 units centered on the network, and subsequently reduced by a ratio of 1/30 upon target onset as a result of saccade preparation toward the target location. Exogenous activity induced by both cues and targets is simulated with a decay constant of, *t_exo_* = −1/55, that degrades the input signal over time. Predictive endogenous orienting evoked by the predictability of cues is simulated using an input signal (*I_endoPred_*) of strength 0.0225 units with a growth constant of, *t_endoPred_* = 1/20, which enhances *I_endoPred_* over time until 620 ms post-cue.

### 4.3. Simulation Procedures

Cue and target exogenous inputs (*I_exo_*) were sent to either the same (cued) or opposite peripheral locations (uncued) after a short interval. Sensory adaptation was activated at the same location as a cue’s exogenous input, briefly reducing subsequent inputs sent to the same cluster of nodes. As a result, a target’s exogenous input strength was reduced by the sensory adaptation function in the cued condition only. When cues were nonpredictive in the 50% predictability condition, no predictive endogenous input was sent to the model when simulating nonpredictive cueing. The predictive endogenous inputs were sent to either the cued or the uncued locations depending on the predictability of the cue. Predictive endogenous inputs were sent to cued locations in 75% predictability conditions and uncued locations in 25% predictability conditions. SRTs for each trial were then calculated as the time difference between target onset and the time when any node activity reached the threshold (80% of maximal firing rate). The accuracy of the model was evaluated using normalized root mean square error (NRMSE) by comparing the difference between empirical and simulated cueing effects. NRMSE values range from 0 to ∞, with a value closer to 0 indicating a better fit (or less residual variance). A NRMSE value of 0 would indicate a perfect fit, and a value of 1 indicates that the simulated data are no better than a straight line at approximating the empirical data.

### 4.4. Simulation Outcomes

When simulating the cued condition of the nonpredictive cueing task, the same nodes were repeatedly stimulated by both cues and targets, activating sensory adaptation that reduces target-induced exogenous inputs at short CTOAs. At long CTOAs, although sensory adaptation is no longer active from 750 ms post-cue onwards, direct inhibition was activated before sensory adaptation dispersed completely, causing cued locations to require more activity to reach the saccade initiation activity threshold. Taken together, simulation results of the nonpredictive cueing task produced ICEs across all CTOAs [*NRMSE* = 0.42], with the strongest simulated ICE of 44 ms at the 600 ms CTOA as a result of sensory adaptation and direct inhibition both being active at this time point. This observation is in general agreement with our behavioral results, in which the strongest ICE of 37 ms was found at 600 ms CTOA as well (see Table 1 and Table 2 for a summary of all behavioral and simulation results).

Simulations of the predictive cueing task included *I_endoPred_* sent to cued locations upon cue onset. This additional input induced by the predictability of cues was able to compensate for the inhibition effects of sensory adaptation and direct inhibition across all CTOAs in the predictive condition [*NRMSE* = 0.63], nullifying all ICEs seen in the nonpredictive cueing simulations. As observed in our behavioral results, no significant cueing effect was found at any CTOAs when cues were predictive. Although the added *I_endoPred_* was able to counteract the inhibition effects, it was insufficient to drive the activity at cued target locations to reach the threshold faster than uncued target locations. In other words, the winner-take-all dynamics between facilitatory and inhibitory components resulted in no ‘winner’ here.

In the simulations of the counterpredictive cueing task, however, *I_endoPred_* was sent to uncued locations due to targets having a lower chance of appearing at cued compared to uncued locations. Added to the inhibition effects at cued locations, which caused slowed activity buildup at the corresponding network nodes in the nonpredictive cueing task, the facilitation effects of *I_endoPred_* caused the activity to build up faster at uncued locations, therefore reaching the required threshold earlier than cued locations. As stated, the uncued location was the winner in the nonpredictive cueing task, and it is an even bigger winner in the counterpredictive cueing task. By taking advantage of *I_endoPred_* received at the uncued nodes, simulated SRTs showed stronger ICEs compared to the nonpredictive cueing task, which closely matched the ICEs found in our behavioral results (*NRMSE* = 0.35).

Although ICEs have been found to be observable for as long as 3 s [45], given the dynamics of brain activity, the strength of direct inhibition should decline gradually to 0, similar to that of sensory adaptation, and not disperse abruptly from its maximum value when direct inhibition stops. However, due to the lack of time course data on ICEs at long CTOAs, our previous model did not account for the fact that direct inhibition (or IOR) does not last indefinitely as we could not estimate the curve parameters of direct inhibition.

The current implementation presents an improved version of our previous model in [6]. The updated model was able to explain the behavioral results of the present study by taking into account the predictability contributed by cues, while keeping the model assumptions and parameters consistent with our previous study.

## 5. General Discussion

The findings of our studies can be summarized as follows: (1) the magnitude of ICEs observed in the three conditions increased consistently in the order of predictive, nonpredictive, and counterpredictive; (2) predictive cueing reduced ICEs observed in the nonpredictive condition; (3) counterpredictive cueing enhanced ICEs observed in the nonpredictive condition; (4) the cue predictability facilitation effects did not become indefinitely stronger at longer CTOAs; and (5) the magnitude of direct inhibition begins to decline at around 1000 ms CTOA.

The time courses of the cueing effects observed in the two studies are consistent with each other. Across all CTOAs (i.e., 300, 600, 900, 1200, and 1500 ms), strong ICEs were observed in the counterpredictive condition as a result of facilitation of uncued targets and inhibition of cued targets, reduced inhibition was observed in the nonpredictive condition due to inhibition of cued targets, and no significant cueing effect was found in the predictive condition due to the facilitatory and inhibitory effects canceling each other out.

In the two studies reported in [6], weaker ICEs at the earliest CTOA of 250 ms compared to longer CTOAs were found, which, we reasoned, were due to lingering cue-elicited activity. This exogenous facilitation effect was not observed in the present studies at the earliest CTOA of 300 ms, when compared to the other effects observed at later CTOAs. Similar patterns of manual RTs were observed by Wright and Richard [9], who found facilitation effects at 66 and 100 ms CTOAs in both the nonpredictive (i.e., 50% cue predictability) and the predictive (i.e., 80% cue predictability) conditions, but only inhibition effects were observed at 300 and 400 ms in the nonpredictive condition. From these and our results, we infer that the facilitation effects observed at very early CTOAs, shorter than approximately 200 ms, may be attributed to the lingering exogenous activity of the cue (see also [46]), which diminishes somewhere between 250–300 ms, leading to observed inhibition effects.

Neurophysiological explanations for our results can be inferred based on previous research, which has found evidence that ICEs are associated with weaker target-related activity in cued conditions as opposed to uncued conditions (e.g., [7,43,44,47]). At a CTOA of 650 ms, single-neuron monkey data from [10] suggested that faster saccadic responses to predictively cued targets with 80% cue predictability were correlated with an increase in activity in the deep layers of the superior colliculus (SC). This finding means that, in the cued condition, the presence of a behavioral facilitation effect is accompanied with an increase in pre-target activity, and the absence of a behavioral facilitation effect also means no such increase in pre-target activity is present. However, in our behavioral studies, this increase in pre-target activity did not lead to faster SRTs to cued targets in the predictive condition, but it nullified all ICEs that were observed in the nonpredictive condition instead. Referring to the hypothesized additive nature of facilitation and inhibition effects, we reasoned that the ICEs observed in the predictive condition are a result of the two opposing mechanisms balancing each other out.

Using a DNF model of the SC, Satel et al. [32] implemented the increase in pre-target activity promoted by predictive cues as a form of endogenous signal that builds up over time at cued locations, demonstrating how it can outweigh inhibition effects and eventually lead to faster SRTs to cued targets. If this predictive endogenous signal can build up at cued locations, we reasoned that it can, too, build up at uncued locations in a counterpredictive condition (i.e., a higher chance of the target appearing at cued rather than uncued location). However, we did not observe ICEs increase as CTOA increases in counterpredictive experiments. In that case, the contribution of predictive endogenous at signals at uncued locations and the lateral interaction of the SC—characterized by short-distance excitation and long-distance inhibition [48,49]—would cause the greater ICEs observed in counterpredictive conditions compared to nonpredictive conditions.

As predictive endogenous activity builds up at uncued locations, neighboring neurons receive strong local excitation (i.e., central zone of facilitation), and further neurons receive lateral inhibition, forming a Mexican-hat-like distribution of neuronal activity [50,51]. Our DNF model used a kernel of connection with short-distance excitation and long-distance inhibition that is commonly referred to as Mexican hat connectivity [41], where neuronal activity is fused together when the spatial distance between the two inputs is close enough, and inputs that are further apart will inhibit one another. Cued locations were further away from uncued locations and within the adjacent zones of inhibited processing, resulting in slower SRTs to cued as opposed to uncued targets. However, we do not know if the strength of the predictive endogenous signal is proportional to the predictive value. Put another way, does a higher cue predictability (e.g., 90%) lead to a stronger facilitation effect? Future research should consider using various predictive values (e.g., 90%, 80%, 20%, and 10%) to better investigate the effects of cue predictability on IOR.

In trials with cue predictability of 25% or 50%, empirical data showed that ICEs were declining in magnitude at CTOAs greater than 900 ms. This trend towards smaller ICEs was unlikely to be caused by sensory adaptation alone as sensory adaptation is relatively weak in late CTOAs, as demonstrated in our simulations, with sensory adaptation configured to decline gradually starting at 400 ms post-cue ([6]; see also [32,34]) and predictions derived from the monkey neurophysiological data (e.g., [43,44,52]). Contrary to these predictions, empirical data in the 25% cue predictability condition showed that ICEs continued to decline at 1200 ms CTOA and beyond. We infer that the declination in ICEs at late CTOAs was caused by the dispersion of direct inhibition, and not sensory adaptation, as demonstrated in the present simulation results.

Aside from sensory adaptation and direct inhibition, there are other, less understood forms of inhibitory mechanisms that can be triggered under certain conditions, such as the late input-based ICE that was observed at a 1050 ms CTOA only when the oculomotor system was suppressed (i.e., a manual response task; e.g., [5]; for a review, see [53]). It is, therefore, worth noting that the series of experiments reported here were designed to examine the inhibitory effects from sensory adaptation and direct inhibition only. Future work should consider examining the role of different response modalities in the time course of facilitatory and inhibitory cueing effects.

Furthermore, although our model does take the input-based and output-based inhibition into account, the function of these mechanisms may not accurately capture the change in magnitude over time, and the experimental design used in the present study does not allow us to disentangle the two effects at early CTOAs. It is, therefore, not clear how much of the resulting overall cueing effect can be attributed to sensory adaptation and direct inhibition, specifically, at each CTOA. To more accurately estimate the function of these individual inhibitory mechanisms in a model, a series of behavioral studies that aim to examine the time course of sensory adaption and direct inhibition by dissociating spatiotopic and retinotopic reference frames will have to be conducted (e.g., [54]).

To conclude, findings from the present studies support our multiple cueing mechanisms theory: (1) the endogenous activity contributed by cue predictability can reduce, or even outweigh ICEs, leading to facilitation effects; (2) cue predictability facilitation effects do not become stronger indefinitely at longer CTOAs; (3) we can exploit the additive nature of facilitatory and inhibitory effects to enhance ICEs by having more uncued than cued trials; (4) temporal predictability is another additive facilitatory endogenous component, along with cue predictability and move signals triggered by target onset; and (5) direct inhibition does not last indefinitely and begins to decay at around 1000 ms post-cue until complete dispersion.

## Figures and Tables

**Table 1:** Mean and (Standard Deviation) SRTs (ms) for each Cueing*CTOA condition with its corresponding cueing effect (cued—uncued) and the percentage of incorrect responses. Values in brackets indicate the simulated values using a DNF model.

Cue Predictability	CTOA	Cued	Uncued	Cueing Effect	Error Rate (%)
75%	300	331.77 (55.79) (293)	322.94 (38.92) (281)	8.82 (12)	0.37
	600	291.13 (45.10) (271)	296.21 (41.31) (269)	−5.08 (2)	0.06
	900	275.93 (47.68) (260)	283.89 (36.95) (268)	−7.97 (−8)	0.21
50%	300	309.44 (36.65) (314)	281.51 (41.18) (281)	27.93 ** (33)	0.19
	600	268.74 (39.59) (313)	231.80 (28.52) (269)	36.94 *** (44)	0.12
	900	259.04 (35.72) (304)	231.17 (26.50) (268)	27.87 *** (36)	0.06
25%	300	310.94 (36.36) (314)	263.56 (37.79) (267)	47.39 *** (47)	0.37
	600	288.33 (37.80) (313)	228.34 (25.75) (242)	60.00 *** (71)	0.12
	900	277.51 (48.87) (304)	221.31 (26.95) (240)	56.20 *** (64)	0.18

* *p* < 0.05, ** *p* < 0.01, *** *p* < 0.001.

**Table 2:** Mean (and Standard Deviation) SRTs (ms) for each Cueing*CTOA condition with its corresponding cueing effect (cued‒uncued) and the percentage of incorrect responses. Values in brackets indicate the simulated values using a DNF model.

Cue Predictability	CTOA	Cued	Uncued	Cueing Effect	Error Rate (%)
75%	900	301.82 (43.81) (260)	289.63 (41.64) (268)	12.19 (−8)	0.06
	1200	266.82 (40.81) (259)	269.49 (52.78) (268)	−2.67 (−9)	0.16
	1500	266.05 (36.87) (254)	258.50 (40.37) (268)	7.56 (−14)	0.19
50%	900	316.02 (41.20) (304)	286.55 (36.90) (268)	29.47 *** (36)	0.06
	1200	277.84 (49.76) (299)	259.20 (38.71) (268)	18.65 ** (31)	0.00
	1500	268.61 (49.02) (290)	250.49 (43.04) (268)	18.12 *** (22)	0.13
25%	900	345.42 (46.92) (304)	286.33 (51.64) (240)	59.09 *** (64)	0.06
	1200	304.49 (59.61) (300)	251.49 (52.86) (240)	53.00 *** (60)	0.03
	1500	282.37 (55.22) (290)	247.77 (47.94) (240)	34.60 *** (50)	0.12

* *p* < 0.05, ** *p* < 0.01, *** *p* < 0.001.
